# Are anti-glutamic acid decarboxylase 65-kDa isoform antibodies related to diabetes or brain tumor?

**DOI:** 10.1186/s40001-022-00674-3

**Published:** 2022-04-06

**Authors:** Buajieerguli Maimaiti, Salamaitiguli Mijiti, Huaiyu Sun, Yinyin Xie, Ting Jiang, Qian Meng, Hongmei Meng

**Affiliations:** Department of Neurology and Neuroscience Center, First Hospital of Jilin University, Changchun, Jilin People’s Republic of China

**Keywords:** Glutamic acid decarboxylase, Malignant glioma, Diabetes mellitus, Stiff-person syndrome

## Abstract

**Background:**

Antibodies against the 65-kDa isoform of glutamic acid decarboxylase (GAD65) are biomarkers of autoimmune disorders and are more common in non-neurological autoimmune diseases than in neurological disorders. As for the central nervous system (CNS), it is well known that GAD65 is primarily associated with stiff-person syndrome, cerebellar ataxia, epilepsy, and paraneoplastic neurological syndrome. However, GAD65 antibodies have not been reported in patients with brain tumors.

**Case presentation:**

This study presents the case of a 62-year-old man who manifested rapidly progressive dizziness with gradually worsening physical disturbance and unstable gait in the 2 months prior to consultation. Antibodies against GAD65 were detected in his serum. Brain magnetic resonance imaging (MRI) showed abnormal signals in the corpus callosum, the semi-oval center in both hemispheres, and the area below the frontal cortex, along with enhanced intracranial lesions in the same regions. Positron emission tomography–computed tomography (PET–CT) showed high metabolism in the corpus callosum, which protruded into both ventricles. Due to signs of malignancy, the patient was diagnosed with a malignant glioma.

**Conclusions:**

This case raises awareness on the fact that anti-GAD65 antibodies may be associated with CNS neoplastic lesions. Early recognition of anti-GAD antibodies could be of great importance for the early diagnosis and targeted treatment of neoplastic lesions, and could lead to better prognosis.

## Introduction

Gamma-aminobutyric acid (GABA) is a major inhibitory neurotransmitter in the central nervous system (CNS), and glutamic acid decarboxylase (GAD) is the rate-limiting enzyme that mediates its synthesis. GAD is a pyridoxal 5’-phosphate-dependent enzyme that is selectively expressed in GABAergic neurons and pancreatic β-cells. Two isoforms with different molecular weights exist, GAD65 and GAD67, which are each encoded by two different genes [1, 2]. GAD65 is a well-recognized major autoantigen in type 1 diabetes mellitus (DM1), and recent studies have shown that high GAD65 antibody serum levels are associated with neurological disorders such as stiff-person syndrome (SPS), cerebellar ataxia, epilepsy, and paraneoplastic neurological syndrome (PNS) [3–6]. According to a meta-analysis, lung and breast tumors are the most frequent lesions associated with PNS manifesting with high anti-GAD65 antibodies [4]. However, no studies have reported a potential correlation between GAD65 and neoplastic CNS diseases. We herein report the case of a 62-year-old man with considerable serum levels of anti-GAD65 antibodies and a cerebral space occupying lesion. In addition, to increase awareness about the neurological syndromes that can manifest with anti-GAD65 antibodies, we have provided a brief review of relevant studies in the field.

## Case description

A 62-year-old man was admitted to our hospital with complaints of rapidly progressive dizziness and unstable gait, which had been present for the previous 2 months. He deviated to the left when walking. The patient had a 10-year history of hypertension and a 1-year history of gout without systematic diagnosis and treatment; he denied having any history of diabetes, autoimmune diseases, blood transfusions, or other familial conditions. Neurological examinations showed no abnormalities, except for spastic paralysis of the left lower limb. It showed decreased muscle strength of the left lower limb (level 4) with hyperactive tendon reflexes. And the Babinski and Chaddock signs were positive on the left side.

**Fig. 1:**
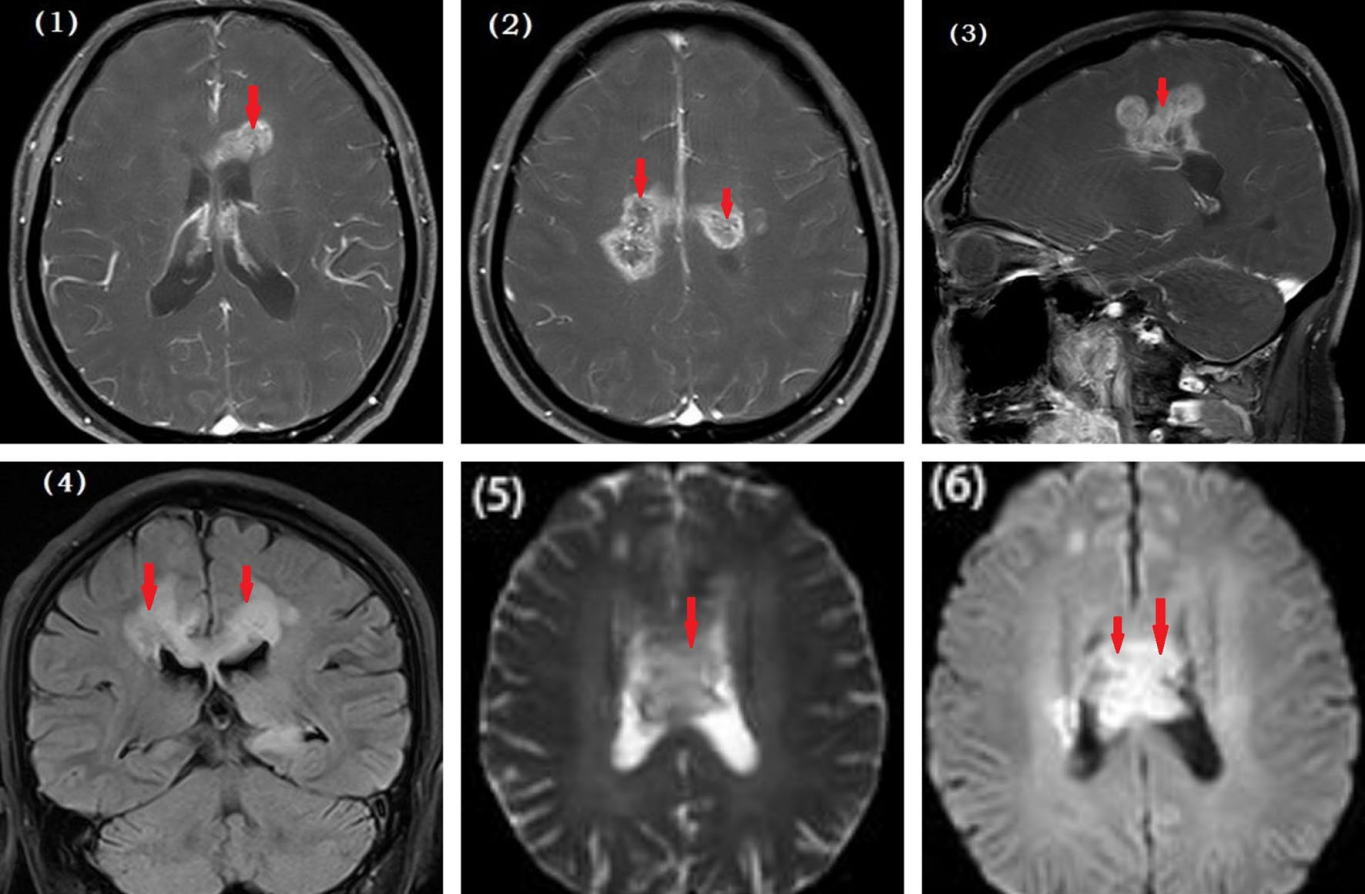
Magnetic resonance imaging (MRI) of the head. (**1–4**) Abnormal lesions are visible in the corpus callosum, semi-oval centers, and the area below the frontal cortex. Contrast-enhanced MRI of the head shows enhanced intracranial lesions within the same areas. The lesions were hyperintense on fluid-attenuated inversion recovery and hypointense on diffusion-weighted imaging (**5, 6**)

**Fig. 2:**
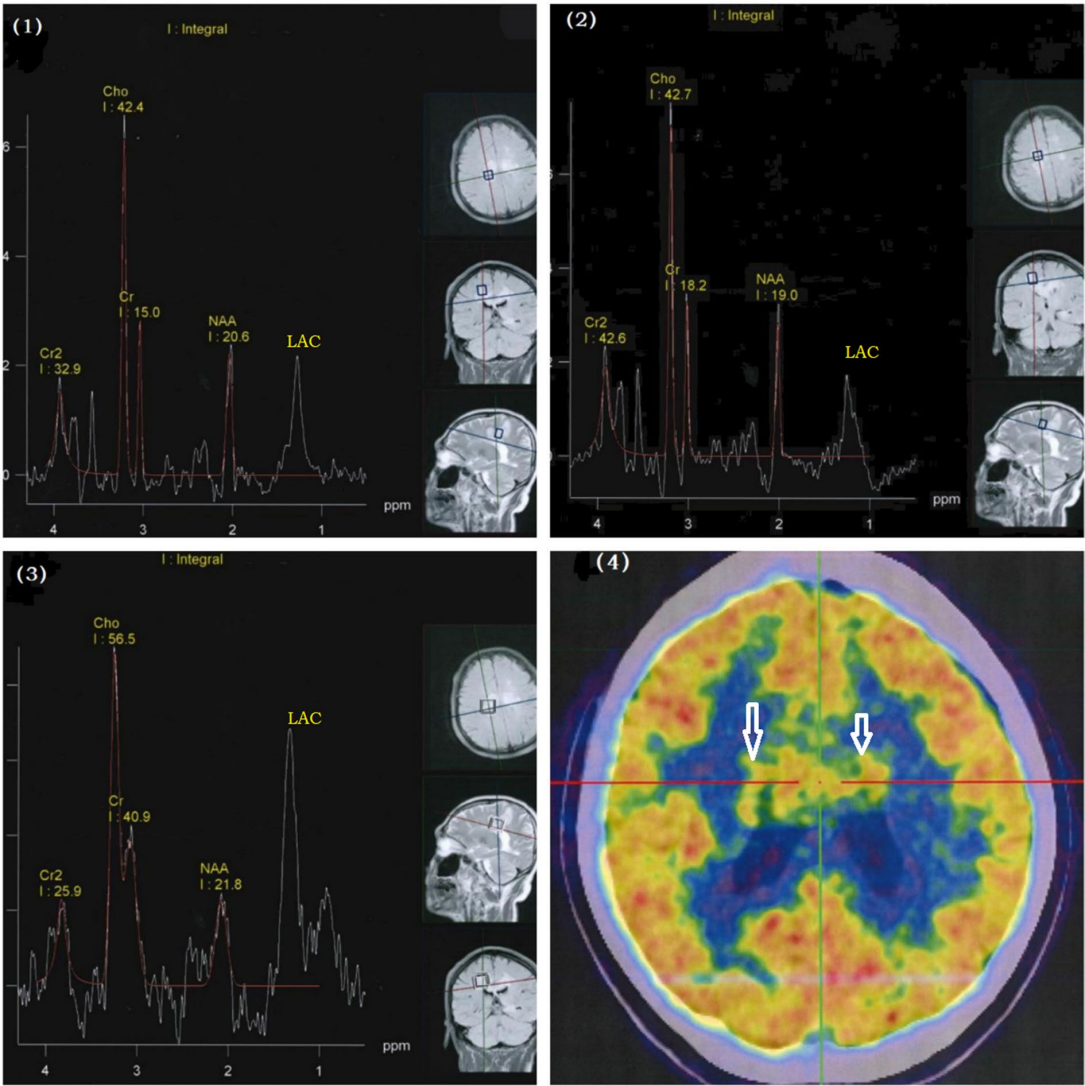
Spectral analysis of lesion metabolites. (**1**–**3**) N-Acetyl aspartate (NAA) and choline (Cho) were decreased and increased, respectively. The Cho + Cr/NAA ratio was 4.47, 3.20, and 2.79 in the corpus callosum, semi-oval centers, and the area below the frontal cortex, respectively. Lactic acid peaks were also increased in these regions. Positron emission tomography–computed tomography (PET–CT). **4** High metabolism was observed in the corpus callosum and both ventricles, suggesting the presence of a malignant glioma

The level of serum glycosylated hemoglobin was 6.20% (reference value: 4.27–6.07). MRI of the head showed abnormal lesions in the corpus callosum, the semi-oval center in both hemispheres, and the area below the frontal cortex with contrast medium enhancement (Fig. 1[1–4]). The lesions were hyperintense on fluid-attenuated inversion recovery and hypointense on diffusion-weighted imaging with aggressive growth and irregular margins (Fig. 1[5–6]). To determine whether the lesions were inflammatory, demyelinating, or neoplastic, we tested for the presence of all the related cerebrospinal fluid (CSF) antibodies. The patient tested positive for anti-GAD65 antibodies. Spectral analysis of lesion metabolites showed that N-acetyl aspartate (NAA) was decreased and choline (Cho) increased (Fig. 2[1–3]). The ratio of Cho and creatine (+ Cr) to NAA (Cho + Cr/NAA) was 4.47, 3.20, and 2.79 in the corpus callosum, semi-oval centers, and the area below the frontal cortex, respectively. Lactic acid peaks were also increased in these regions. Positron emission tomography–computed tomography (PET–CT) showed high metabolism in the corpus callosum and both ventricles, suggesting the presence of a malignant glioma (Fig. 2[4]).

During hospitalization, the condition of the patient deteriorated rapidly, and, unfortunately, due to the family’s economic situation, he decided not to undergo stereotactic brain biopsy and forfeited further treatment.

## Discussion

GAD is present in regions of the neural tissue that comprise inhibitory interneurons as well as in pancreatic islet β-cells [7]. Its two isoforms, GAD65 and GAD67 [1], have different enzyme activity. Located in the cytoplasm, GAD67 is constitutively active and provides a steady source of GABA. On the other hand, GAD65 is mainly located in synaptic vesicles, is auto-inactivated during enzyme activity, and is primarily present as an apoenzyme, providing a boost in GABA production under conditions that require rapid surges of neurotransmitter synthesis and release [8]. GAD65 is a major autoantigen in patients with DM1, and the rate of positivity to anti-GAD antibodies in pre-diabetic stages and patients with DM1 is 70%–90% [2]. The latent autoimmune diabetes (LADA) in adults is a special type of DM1, which is an autoimmune disease. Due to its insidious onset and slow progress, as well as the leftover islet-β cell function and rapid oral hypoglycemic drug effectiveness, the LADA can be easily misdiagnosed as type 2 diabetes. However, the simultaneous detection of GAD and anti-islet cell antibodies helps in the early identification of LADA [9]. In the patient herein presented, the level of serum glycosylated hemoglobin was slightly higher than reference values. The patient also had glucose in urine. Therefore, we believe that the serum GAD65 antibody levels that were detected could possibly be attributed to a pre-diabetic stage or late-onset type 1 diabetes.

Recent research has concluded that high anti-GAD65 antibody levels in the serum (titers > 20 nmol/L) or CSF are associated with various autoimmune neurological diseases, including autoimmune epilepsy, SPS, cerebellar ataxia, limbic encephalitis, progressive encephalomyelitis with rigidity and myoclonus, and PNS [10]. Representative neurological syndromes in patients with high anti-GAD antibody levels are shown in Table 1. Anti-GAD65 antibodies in patients with neurological disorders are reportedly associated with small-cell or non-small cell lung cancer, other neuroendocrine lineage neoplasms, testicular seminoma, thymoma or thymic carcinomas, thyroid neoplasia, adenocarcinomas of the breast, gastrointestinal tract, and kidney, lymphomas, and myeloma [11]. Cancer risk may increase with age and is higher in men and patients with concomitant antibodies against neuronal cell-surface antigens [4]. It has been suggested that patients with high anti-GAD antibody levels and classic PNS or neurological syndromes that are not typically associated with anti-GAD antibodies should be screened for cancer. Considering the tumors identified in this study and previously reported cases, tumor workups should include mammograms, serum tumoral marker tests, and chest PET–CT or CT, depending on the clinical setting. The specific correlation mechanism between GAD65 antibodies and the abovementioned tumors is unclear.

**Table 1:** GAD65 autoimmune neurological associations

Stiff-person syndrome (classic form, limited form, and progressive encephalomyelitis with rigidity and myoclonus)
Cerebellar ataxia
Intractable epilepsy (autoimmune)
Brain stem syndrome
Extrapyramidal syndromes
Corticospinal spasticity
Limbic encephalitis
Peripheral neuropathies
Ocular features (nystagmus and myoclonus features)
Myelopathy
Autonomic neuropathy
Paraneoplastic neurological syndrome includes small-cell or non-small cell lung cancer, other neuroendocrine lineage neoplasms, testicular seminoma, thymoma or thymic carcinomas, thyroid neoplasia, adenocarcinomas of the breast, gastrointestinal tract, and kidney, lymphomas, and myeloma

In this study, we performed chest CT scans and whole-body PET–CT scans to diagnose GAD65-related tumors and exclude the possibility of brain metastasis. The PET–CT results in the lungs, extremities, and abdomen were negative, brain PET–CT showed high metabolism in the genu of the corpus callosum and both ventricles, implying the presence of a malignant glioma.

The differential diagnosis for a brain lesion relies on a patient’s age, risk factors for infection, the presence of other tumors, and imaging characteristics. Primary differential diagnoses include metastases, infections, inflammatory disorders, and vascular lesions. A well-defined lesion with a normal overlying cortex tends to metastasize in a single ring-enhanced lesion. Lesions with ill-defined contours and adjacent cortical enlargement tend to be high-grade gliomas, while lesions with reduced central diffusion are more likely to be abscesses [12]. The final diagnosis was consistent with a malignant glioma based on the abovementioned imaging results. However, no research has reported an association between GAD65 antibodies and gliomas thus far.

As the most common primary brain tumor, gliomas are all the tumors that originate from glial cells. Gliomas constitute approximately 27% of all primary CNS tumors and 80% of malignant tumors [13]. Like many other cancers and rapidly proliferating cells, gliomas often metabolize glucose into lactate, even when oxygen is present; this is known as the Warburg effect, and it enables tumor cells to use glucose-derived carbons to synthesize essential cellular ingredients while still generating sufficient adenosine triphosphate to fuel cellular reactions. Hyperglycemia conventionally increases the tumor growth rate in animals and humans [14]. Seyfried et al. showed that blood glucose is directly correlated with in vivo glioma growth [15]. Glucose uptake is a rate-limiting glucose metabolism step mediated by glucose transporter (GLUT) proteins. GLUT expression is consistently elevated in cancer cells of varying tissue origins, and various significant oncogenes conventionally directly upregulate GLUT and glycolytic proteins. These oncogenes include the MYC (bHLH transcription factor), KRAS (GTPase), and B-Raf (serine/threonine kinases) proto-oncogenes. Among the 14 known GLUT isoforms, GLUT 1 and 3 overexpression are most often associated with malignant transformation and progression in gliomas and other tumors, and both have been correlated to a poor clinical prognosis [16].

Based on the diagnosis of malignant glioma, we believe that another possible reason for the increase in serum anti-GAD65 antibody levels observed in our patient could be the overexpression of the abovementioned proto-oncogenes, which can lead to increased GLUT expression. The responsive rise in blood glucose further promotes the function of pancreatic islet β-cells, which consequently heightens blood GAD65 antibody levels.

## Conclusion

Researches have shown that high GAD65-ab serum levels are associated with neurological disorders and cancers in lung or breast, but no studies have reported a potential correlation between GAD65 and neoplastic CNS diseases. This case raises awareness on the fact that anti-GAD65 antibodies may be associated with CNS neoplastic lesions. Further research is needed to confirm this conclusion. However, we emphasize the importance of clinical screening for patients with progressive dizziness and gait ataxia. Specifically, anti-GAD antibody levels should be tested, and, if present, should raise suspicion of an underlying tumor in the CNS.

## Data Availability

All relevant raw data in this study will be freely available to any scientist wishing to use them without breaching participant confidentiality for non-commercial purposes.

## Abbreviations

Cho: Choline
CNS: Central nervous system
Cr: Creatine
CSF: Cerebrospinal fluid
DM1: Type 1 diabetes mellitus
GABA: Gamma-aminobutyric acid
GAD: Glutamic acid decarboxylase
GLUT: Glucose transporter
LADA: Latent autoimmune diabetes
NAA: N-Acetyl aspartate
MRI: Magnetic resonance imaging
PET–CT: Positron emission tomography–computed tomography
SPS: Stiff-person syndrome
